# The significance of Mg in prebiotic geochemistry

**DOI:** 10.1111/j.1472-4669.2012.00323.x

**Published:** 2012-07

**Authors:** N G Holm

**Affiliations:** Department of Geological Sciences, Stockholm UniversityStockholm, Sweden

## Abstract

Magnesium plays a special role in biochemistry because of its ability to coordinate six oxygen atoms efficiently in its first coordination shell. Such oxygen atoms may be part of one or two charged oxyanions, which means that Mg^2+^ can, for instance, tie together two different phosphate groups that are located at distance from each other in a macromolecule, and in this way be responsible for the folding of molecules like RNA. This property of Mg^2+^ also helps the stabilization of diphosphate and triphosphate groups of nucleotides, as well as promoting the condensation of orthophosphate to oligophosphates, like pyrophosphate and trimetaphosphate. Borates, on the other hand, are known to promote the formation of nucleobases and carbohydrates, ribose in particular, which is yet another constituent of nucleotides. The oldest borate minerals that we find on Earth today are magnesium borates. Dissolved borate stabilizes pentose sugars by forming complexes with cis-hydroxyl groups. In the furanose form of ribose, the preferential binding occurs to the 2 and 3 carbon, leaving the 5 carbon free for phosphorylation. The central role of Mg^2+^ in the function of ribozymes and its ‘archaic’ position in ribosomes, and the fact that magnesium generally has coordination properties different from other cations, suggests that the inorganic chemistry of magnesium had a key position in the first chemical processes leading to the origin and early evolution of life.

## Introduction

### Magnesium in the Earth system

Magnesium (Mg) is a common element on Earth and the other terrestrial planets. It is one of the eight main elements of Earth’s crust and one of the four major elements making up the mass of the whole Earth. Furthermore, Mg is one of the principal constituents of silicate minerals that build up Earth, like olivine, pyroxenes, and Mg layer silicates (e.g., serpentines, talc, Mg smectites). The concentration of divalent magnesium (Mg^2+^) in contemporary ocean water is 52.8 mmol kg^−1^. The coordination geometry of magnesium is normally octahedral, that is, the Mg atom coordinates six atoms – almost always oxygen – around itself in its first coordination shell (Kehres & Maguire, 2002). Six-coordinated Mg^2+^ has a small ionic radius of 0.65 Å, at the same time as it has the largest hydrated radius of any common cation. The volume difference between hydrated and ionic Mg^2+^ is almost 400-fold (Kehres & Maguire, 2002). In the marine geochemical environment, magnesium is particularly important because the tri-octahedral layer of the common smectites in sediments consists primarily of brucite, the mineral name of magnesium hydroxide (Mg(OH)_2_). The ocean floor beneath the sediment layers consists of basalts and ultramafic rocks that have a high content of primary ferromagnesian silicate minerals (olivine and pyroxenes). Alteration of these minerals in contact with water leads to ‘serpentinization’, a process in which olivine and pyroxenes are transformed to serpentines. Serpentines like lizardite cannot accommodate all of the magnesium of the primary minerals, so brucite is formed as a separate mineral phase, often in veins of the serpentine, at temperatures below about 315 °C (Holm & Neubeck, 2009). Laboratory experiments of olivine alteration by our own research group show a spike of dissolved Mg^2+^ at about 25 ppm when the olivine surface is fresh and then a strong decrease in the concentration of the fluid phase with nucleation and precipitation of secondary Mg phases, such as brucite (Neubeck, 2011).

Brucite may be transformed into double-layer hydroxides (DLH), also called layered double hydroxides (LDH), if a fraction of the divalent Mg^2+^ is replaced by common trivalent cations such as Al^3+^, Fe^3+^, and Cr^3+^ (Arrhenius *et al.*, 1997).

## Magnesium as a Catalyst

**Fig. 1:**
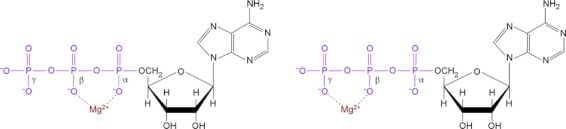
Possible Mg^2+^ complexes with ATP. The most common complex is formed with oxygen of the β- and γ-P parts of the oxyanion (courtesy J. Cronk).

Magnesium ions play critical roles in cellular metabolism. They stabilize structures of proteins, nucleic acids, and cell membranes by binding to the macromolecule’s surface (Yang *et al.*, 2004). They are also key to enzymatic reactions in various ways; for instance, they can generate magnesium-substrate scaffolds to which enzymes bind. However, the best known example of divalent Mg in life processes is probably the central role that it has in photosynthesis as a component of chlorophylls. Anoxygenic photosynthesis occurred by photosynthesizing anaerobic bacteria already before the existence of cyanobacteria (Olson, 2006). Reported evidence suggests that the photosystem in the anoxygenic purple photosynthetic bacteria is the most ancient known (Blankenship, 2001). However, divalent magnesium has many more functions in biological systems; of the many metal cations that form complexes with nucleotides, nucleic acids and enzymes, perhaps none is more essential than Mg^2+^ (LaRowe & Helgeson, 2006; Freisinger & Sigel, 2007). According to the ‘RNA World’ hypothesis, the first enzymes – the ribozymes – consisted of ribonucleic acid (RNA), which depended on Mg^2+^ for its self-cleavage (Gilbert, 1986; Cech, 2002). Divalent magnesium is also present in all deoxyribonucleic acid (DNA) and RNA activation processes (Anastassopoulou, 2003). These circumstances point to a central role for Mg in the geochemistry that presumably led to the first life-like processes. Maguire & Cowan (2002) have reviewed the unique properties of Mg^2+^ among biologically relevant cations. The Mg^2+^ is a cofactor of enzymes and an essential catalyst for many biochemical reactions (Yamagata *et al.*, 1995). For instance, polyphosphate generation often has an absolute requirement for divalent metal ions, typically Mg(II) (de Zwart *et al.*, 2004). It is also well known that Mg^2+^ is required for the stabilization of the diphosphate group of adenosine diphosphate (ADP) and the triphosphate group of adenosine triphosphate (ATP) (Lindh, 2005). The reason is that Mg^2+^ forms six-membered rings with the oxygen and phosphorus atoms of ADP and ATP (Fig. 1). In solution, complex formation with the β- and γ-P of ATP is favored (Lindh, 2005). Divalent magnesium is also known to facilitate the stacking of adenosine monophosphate (AMP), which may promote oligomer formation (Herrero & Terrón, 1998). The intra- and extracellular concentration of Mg^2+^ in organisms is about 1 mmol kg^−1^ (Grubbs, 2002; Lindh, 2005). Cytosolic (intracellular fluid) ATP binds up to 50% of total cellular Mg^2+^ in virtually all cells (Maguire & Cowan, 2002). Divalent magnesium in aqueous solution can form the deprotonated Mg(OH)(aq)^+^ complex that still binds to nucleotides like RNA (Walter & Engelke, 2002). It has been shown by several investigators that magnesium pyrophosphate (MgPPi) is easily formed under mild abiotic hydrothermal conditions (165–180 °C) from magnesium salts and orthophosphate (Pi) (Seel *et al.*, 1985, 1986; Kongshaug *et al.*, 2000). PPi formation is promoted by low activity of water in the system, for instance through low water-to-rock ratio (Russell & Hall, 1997). Seel and co-workers instead used magnesium monohydrate phosphate dispersed in water (saturated solution) in their syntheses, whereas Kongshaug and co-workers obtained low water activity by the use of phosphoric acid. Hermes-Lima & Vieyra (1992) were successful in condensing pyrophosphate (PPi) from orthophosphate at room temperature in the presence on a fresh precipitate labeled as ‘magnesium phosphate’. To reduce the activity of water, they replaced 80% of the water by dimethyl sulfoxide. Pyrophosphate formation was shown to be most efficient above pH 9. However, because the method that they specified involved high pH, it is likely that this ‘magnesium phosphate’ that they produced was actually brucite or a mix of brucite and protonated Mg-phosphate, in analogy to that produced for scavenging of low concentrations of phosphate in ocean water by the procedure of Karl & Tien (1992).

## Condensed Phosphates and Their Role in Biochemistry

A major reason why nature has chosen phosphate for so many purposes in living systems is that it can link up to two adjacent groups, like in nucleic acids, and still remain ionized (Westheimer, 1987). For this purpose, the linking ion has to be at least trivalent. Therefore, phosphate derivatives like pyrophosphate (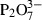), complexed to Mg^2+^, can be a ubiquitous leaving group in biochemistry.

**Fig. 2:**
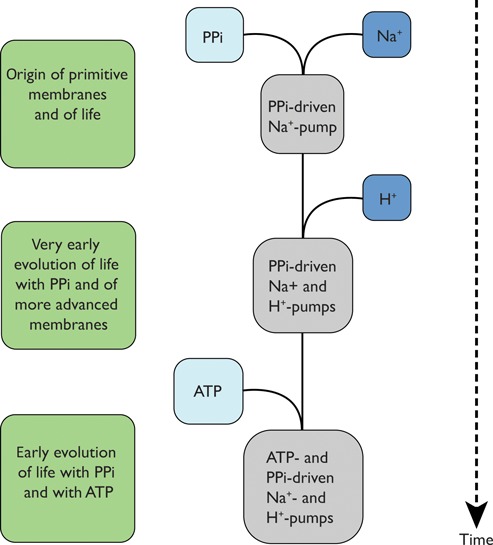
Evolutionary scheme for cation pumping through membranes (modified from Holm & Baltscheffsky (2011).

Mulkidjanian *et al.* (2008) have suggested an earlier transport of Na^+^ over H^+^ by pyrophosphatases (PPases) through biomembranes. Membrane PPases couple the hydrolysis of inorganic pyrophosphate to the active transport of cations across membranes. The PPases always require Mg^2+^ for function (Baykov *et al.*, 1993; Luoto *et al.*, 2011). The hyperthermophilic bacterium *Thermotoga maritima*, found in hydrothermal environments, as well as the mesophile *Methanosarcina mazei* contain membrane-bound Na^+^-pyrophosphatases (Tm-PPase and Mm-PPase, respectively) that are homologs to H^+^-PPases (Belogurov *et al.*, 2005; Malinen *et al.*, 2008). Both Tm-PPase and Mm-PPase have an absolute requirement for Na^+^ as well as Mg^2+^, but display maximal activity in the presence of millimolar levels of K^+^. Holm & Baltscheffsky (2011) have, therefore, proposed that PPi preceded ATP and Na^+^ transport preceded the H^+^ transport in connection with the origin and initial evolution of life on Earth (Fig. 2).

It has been known for some time that hydrogen containing magnesium phosphates like whitlockite (Ca_18_Mg_2_H_2_(PO_4_)_14_ and newberyite (MgHPO_4_·3H_2_O), that is, protonated orthophosphates, at heating react to form condensed phosphates and water (Sales *et al.*, 1993; Gedulin & Arrhenius, 1994; Arrhenius *et al.*, 1997). The phosphate condensation is attributed to the protonation of the phosphate, because at heating, the hydrogen reacts with one of the oxygen ligands of the phosphorus and leaves as water. As a response, the structure of the orthophosphate (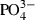) rearranges to form one or more anhydride P–O–P bonds (Arrhenius *et al.*, 1997), that is, the backbone of condensed phosphates. Experiments with newberyite have revealed that dehydration of this crystalline mineral on heating results in an amorphous, more soluble, phase (Sales *et al.*, 1993). During the initial stages of the dehydration process (100–300 °C), condensation to pyrophosphate occurred. At about 200 °C, triphosphate (PPPi) started to appear. However, at 300 °C, a decrease in the concentration of pyrophosphate began, whereas the concentration of triphosphate continued to increase to at least 400 °C, that is, it reaches its maximum in the temperature interval in which dehydration of a subducting slab in a convergent plate margin occurs, that is, 300–375 °C (Alt & Shanks, 2006; Holm & Baltscheffsky, 2011).

A related way of PPi formation can be found in the oxidation of the phosphide mineral schreibersite (Fe,Ni)_3_P. This mineral is normally referred to as a component of iron meteorites (Pasek, 2008), but it is also known to occur in terrestrial basalts (Pauly, 1969; Ulff-Møller, 1985), as well as an indigenous mineral in lunar basalts (El Goresy *et al.*, 1971). The schreibersite appears to be formed as a by-product to phosphoran olivine in P-rich basalt melts at fast quenching (Boesenberg & Hewins, 2010), and it is possible that the occurrence of this compound is the solution to the ‘phosphate problem’ as discussed by Schwartz (1971, 2006) and Rauchfuss (2008), that is, dissolution of phosphate compounds is necessary before activation can occur. Schreibersite oxidizes slowly in contact with fluid water as the surrounding mineral matrix gets weathered, and forms several phosphorus species of mixed oxidation states like orthophosphate, pyrophosphate, hypophosphate, phospite, etc. (Pasek & Lauretta, 2005; Pasek *et al.*, 2007, 2008; Pasek, 2008). In systems containing dissolved Mg^2+^ and Ca^2+^ chloride salts whitlockite is also formed (Pasek & Lauretta, 2005).

Trimetaphosphate (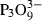) has been shown to be the most effective condensing agent among polyphosphates, particularly because it can serve as a phosphorylating reagent in aqueous and strongly alkaline solution (pH 12) (Etaix & Orgel, 1978; Yamagata *et al.*, 1995; Ozawa *et al.*, 2004). The prebiotic synthesis proceeds by the thermolysis of phosphate to yield linear polyphosphates, which in turn cyclize to trimetaphosphate in aqueous systems (Kalliney, 1972; Ferris & Usher, 1988). Twenty years ago, Yamagata *et al.* (1991) published a paper on the occurrence of condensed phosphates in fumaroles of the Mount Usu volcano in Japan. The phosphates were believed to have been formed through partial hydrolysis of P_4_O_10_. Approximately equal concentrations of pyrophosphate (0.45 μm) and triphosphate (0.37 μm) were found. It is not known whether this was cyclic or linear triphosphate, but in the case of linear triphosphate, it would have cyclized to trimetaphosphate in water.

Decyclization of trimetaphosphate into linear triphosphate, on the other hand, requires the addition of Mg^2+^ (Turian & Rivara-Minten, 2001). Mechanistically, this can be explained by the shielding of 2+1 PO^−^ groups by two Mg^2+^, keeping one positive charge free to ionize H_2_O to H^+^ and OH^−^, which would allow nucleophilic attack of one of the three P–O–P anhydride bonds by the OH^−^ (see section ‘Magnesium, nucleotides, and folding’ below). Linear triphosphate is an extremely important constituent of nucleotides in the sense that RNA chains always start with guanosine triphosphate (pppG) or adenosine triphosphate (pppA).

## Magnesium, Nucleotides, and Folding

**Fig. 3:**
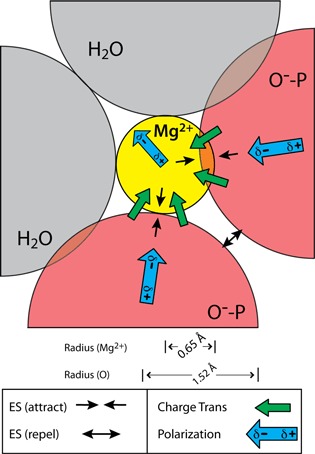
Interactions of a hexacoordinated magnesium ion with two anionic oxygen atoms and four water molecules (only the two in-plane water molecules are shown). Reprinted with permission from Petrov *et al.* (2011). Copyright 2011 RNA Society.

Magnesium plays a special role in the function and folding of nucleotides (Freisinger & Sigel, 2007). As the coordination geometry of Mg^2+^ is octahedral, it may coordinate six oxygen atoms of different oxyanions. The divalent Mg ion has an enhanced ability compared to other cations to form bidentate clamps with RNA, because the oxyanions of RNA phosphates have significant affinity for Mg^2+^ (Fig. 3) (Petrov *et al.*, 2011). Unlike other cations, Mg^2+^ may bring oxygens from two different (charged) phosphate groups in its first shell into direct contact with each other (Hsiao & Williams, 2009). The coordination capacity of Mg^2+^ is attributed to its size, which makes it possible to simultaneously coordinate negatively charged oxygen of two phosphate groups (Yamagata *et al.*, 1995). Divalent magnesium therefore normally coordinates two adjacent oxygen atoms of pyro- and triphosphate. This is also the reason why, during RNA-folding, Mg^2+^ can offset the phosphate–phosphate repulsion by being in a central position between the two groups. For the same reason, Mg^2+^ has been shown to be an extremely effective catalyst for the synthesis of 2′,3′-cyclic AMP and ATP in aqueous solution (Yamagata *et al.*, 1995). Divalent magnesium was shown to accelerate this reaction approximately one hundred times more compared with systems with other cations, like Ca^2+^ (ibid.). The difference in this respect between Mg^2+^ and Ca^2+^ is attributed to the difference in ionic radius. The smaller magnesium ion (0.65 Å, coordination number CN = 6) can form a complex with two or three oxygen atoms of different phosphate groups in a phosphate ester more strongly than a divalent calcium ion (0.99 Å, CN=6) (Yamagata *et al.*, 1995).

Ozawa *et al.* (2004) have shown that phosphorylation of AMP into ADP and ATP can outrun their hydrolysis in a hydrothermal environment (100–130 °C) if trimetaphosphate is used as the phosphate source. The degree of phosphorylation showed a strong positive correlation to an increase in Mg^2+^ activity. The recovery of ATP was higher than ADP at 100 °C, while the opposite was true at 130 °C.

Another effect of magnesium’s ability to coordinate oxygen in phosphate groups is that orthophosphate is a strong inhibitor of brucite dissolution at pH>10.5 (Pokrovsky *et al.*, 2005). Brucite may quantitatively co-precipitate orthophosphate, ATP, and most other phosphorylated organic compounds (Karl & Tien, 1992). In DLH, which derive from brucite, anions of interest in the context of molecular evolution have a sequence of binding energies Cl^−^ < 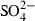 < 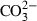 < 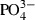 < linear oligophosphates (like PPPi)<cyclic oligophosphates (like trimetaphosphate) (Arrhenius *et al.*, 1997). Choy *et al.* (1999) have demonstrated that nucleotides such as CMP, AMP, GMP, and even DNA can hybridize with DLH, giving rise to a heterostructured nanohybrid which consists of one nucleotide layer and Mg(OH)_2_ alternatively. The nucleotides stabilized in the interlayer space of DLH retain their chemical and biological integrity. Thermal stability of the encapsulated nucleotides up to 300 °C has been observed (Choy *et al.*, 2004).

## The Chemistry of Nucleotides May Include Borate as an Active Compound

Saladino *et al.* (2011) have recently shown that formamide, the hydrolysis product of HCN, oligomerizes in the presence of borate minerals yielding, at the same time, nucleobases and carboxylic acids. Their experiments were carried out at 160 °C in the presence of about 20 different borate minerals and thus yielded the building blocks of both the genetic and metabolic apparatuses. Particularly, the Na-Mg-Al borate mineral dravite (NaMg_3_Al_6_(BO_3_)_3_Si_6_O_18_(OH)_4_) was shown to be efficient in promoting the formation of purine (19% recovery). The efficiency in purine formation in the presence of dravite was probably due to the oligomerization of HCN to its tetramer diaminomaeleonitrile (DAMN), which was shown to be present in the experimental system. DAMN is an intermediate in the abiotic formation of purines, pyrimidines, as well as amino acids from HCN (Ferris & Usher, 1988). Hydrogen cyanide, in turn, may be formed from ammonia and methane or carbon monoxide in oceanic basement and then be concentrated on secondary mineral surfaces or as ferrocyanide (Holm & Neubeck, 2009).

Boron has never been in focus of geobiology because it is not a major component of biological macromolecules. Boron has, however, strong affinity for organic material because it forms trigonal and tetrahedral complexes with oxygen groups. Borate readily forms complexes with a wide variety of sugars and other compounds containing cis-hydroxyl groups, such as humic substances (Bolaños *et al.*, 2004; Panagiotopoulos & Sempéré, 2005). Seawater is the source of almost all borate in altered oceanic crust. In this sense, it is similar to phosphorus.

## Prebiotic Synthesis and Stability Conditions of Nucleotide Constituents

LaRowe & Regnier (2008) have calculated the thermo-dynamic potential for abiotic synthesis of the five common nucleobases (adenine, cytocine, guanine, thymine, and uracil) and the two monosaccharides (ribose and deoxyribose) in RNA and DNA from the precursor molecules CH_2_O and HCN. The temperature, pressure, and bulk composition conditions were chosen to be representative for hydrothermal conditions in ultramafic settings like the Lost City hydrothermal system (Delacour *et al.*, 2008). All of these biomolecules were shown to be thermodynamically favored to be synthesized throughout the temperature range from 0 °C to between 150 and 250 °C, depending on the single biomolecule.

Mechanistically, Yuasa & Ishigami (1977) have shown that periclase (MgO) and magnesite (MgCO_3_) serve as deprotonating reagents for the condensation of HCN to DAMN. At least systems with MgO proceed with further reactions to amino acids and heterocyclic nitrogen compounds like adenine and 4-aminoimidazole-5-carboxyamide (AICAI). In this context, it is notable that Ferris and co-workers (see, for instance, Ferris, 1984) have worked out a prebiotic synthesis pathway from DAMN via AICAI to a wide array of purines, including adenine and guanine.

Pentoses, like ribose, can be formed by the formose reaction under strongly alkaline conditions from simple organic precursors (formaldehyde and glycolaldehyde) (Ferris, 2005). For a while, the formose reaction was an outdated concept in prebiotic chemistry because the reaction was considered to be non-selective. However, it is now believed by many scientists that a selective mechanism that favors the formation of ribose has been identified (see below). The formation of monosaccharides proceeds by the stepwise condensation of formaldehyde to a dimer (glycolaldehyde), trimer, etc. Aldehydes can be formed directly from elemental carbon in the presence of water (Flanagan *et al.*, 1992). Elemental carbon in the form of graphite and amorphous carbon is common in peridotites (Delacour *et al.*, 2008). The initial reaction of elemental carbon with water gives hydroxymethylene, which can rearrange to formaldehyde. A new hydroxymethylene molecule can then add onto the formaldehyde (as well as larger aldehyde molecules) and form glycolaldehyde.

Delidovich *et al.* (2009) have carried out condensation reactions of monosaccharides with formaldehyde, glycolaldehyde, and glyceraldehyde in, what they call, ‘a weakly alkaline medium’ (pH 10.4) at 70 °C in the presence of MgO as a catalyst. The condensation of glycolaldehyde and formaldehyde in the presence of the hydrated MgO at pH 10.4 resulted in the formation of trioses, tetroses, and pentoses.

It has been shown that borate minerals stabilize ribose and simultaneously favor its five-membered furanose ring (Prieur, 2001; Ricardo *et al.*, 2004; Amaral *et al.*, 2008). As previously mentioned, borate readily forms complexes with a wide variety of sugars and other compounds containing cis-hydroxyl groups (Bolaños *et al.*, 2004; Panagiotopoulos & Sempéré, 2005). Once formed, the cyclic form of the pentose like ribose forms a stable, less reactive complex with borate. The binding preferences of borate to pentoses has been determined to be ribose>lyxose>arabinose>xylose (Li *et al.*, 2005). NMR analysis shows that borate occupies the 2 and 3 positions on the furanose form of ribose, thus leaving the five position available for potential reactions like phosphorylation (Benner, 2004; Ricardo *et al.*, 2004). Borate-bound ribose is formed preferentially at pH>9, because the predominant boron species at lower pH is boric acid (Amaral *et al.*, 2008). It has also been shown to be stable for long periods of time at temperatures of about 60 °C (ibid.). In biological systems, the purine nucleotides are synthesized by constructing the purine base on a pre-existing ribose-5-phosphate (Joyce, 1989). However, results by Etaix & Orgel (1978) show that adenosine-5′-triphosphate can be synthesized directly from adenosine and trimetaphosphate at pH 12 if the 2 and 3 OH groups of the ribose are blocked by borate.

Prieur (2009) has shown that it is possible to phosphorylate pure ribose with trimetaphosphate to ribose-5-phosphate in the presence of borate salts. According to Prieur, pyrophosphate does not appear to have the same phosphorylating effect. At high pH (pH>10), the nitrogen in the N9 position of adenine loses a proton so that adenine becomes an anion (Gonella *et al.*, 1983). The adenine anion may react with the C1 position of a pentose-5-phosphate in the open form with the aldehyde group exposed to attack by a nucleofile and form a β-glycosidic bond (Mellersh & Smith, 2010). In order then to stabilize the product, a furanose or pyranose ring needs to be formed, so only pentoses or longer carbohydrates could participate in this type of reactions. This pH effect may support a notion that prebiotic purine nucleotides were, in principle, synthesized in the same way like in modern biological systems by constructing the purine base on a pre-existing ribose-5-phosphate (Holm *et al.*, 2010).

## Ribozymes and Ribosomes

Ribozymes are ribonucleic acid enzymes that are capable of both catalyzing chemical reactions and storing genetic information (Cech *et al.*, 1981; Gilbert, 1986). These two features support the hypothesis of a pre-protein RNA world (Gilbert, 1986; Bereźniak *et al.*, 2010). In the first report on catalytic RNA (or a ribozyme) by Thomas Cech’s group, incubation of the pre-rRNA had been carried out in the presence of Mg^2+^ (Kruger *et al.*, 1982; Cech, 2002). A principal feature of RNA is its high negative charge density. Therefore, the presence of positively charged ions is essential for the structural integrity and catalytic activity of all ribozymes, as well as for their cleavage (Sreedhara & Cowan, 2002; Walter & Engelke, 2002; Lindh, 2005). In the cell, the favorite cation of choice is Mg^2+^, as it has such high affinity for the negatively charged backbone of RNA and is the most abundant divalent metal ion.

The ribosome, which synthesizes proteins in cells, is one of the most ancient molecular machines in all living systems. It is believed to have achieved its present form before the emergence of the last common ancestor of modern life (Fox & Naik, 2004). The ribosome is actually a large ribozyme in which the catalytic center is still composed of RNA (Walter & Engelke, 2002). It has been known for decades that Mg^2+^ is involved in both the structure and catalytic activity of ribosomes (Klein *et al.*, 2004; Lindh, 2005). The core region of the ribosomal large subunits (LSUs), defined with the site of peptidyl transfer (PT) as the origin, is characterized by the greatest Mg^2+^ density and the lowest ribosomal protein density (Hsiao *et al.*, 2009). As one moves from the center to the periphery of the LSU, proteins replace magnesium ions. Near the PT-origin, phosphate oxygens more frequently act as inner sphere Mg^2+^ ligands (Hsiao *et al.*, 2009). It is, therefore, tempting to interpret the density distribution of Mg(II) as a record of chemical evolution of this ancient cellular machinery. Divalent magnesium would have been responsible for the functional folding of early RNA and has successively over time been replaced for the same purpose by the more sophisticated proteins.

## High pH in Plate Tectonic Convergent Margins

**Fig. 4:**
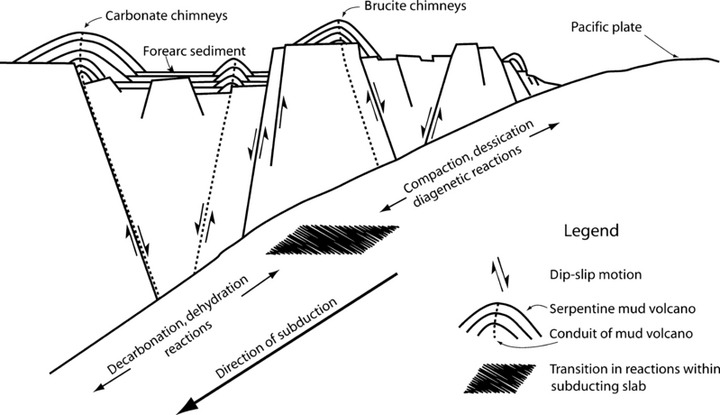
Schematic cross-section of typical settings for mud volcanoes in the Mariana forearc. Mud rises along fault-controlled conduits and volcanoes form mainly at tops or bases of horst blocks, or along faults. During early subduction, the descending plate is heated and dehydrated. Adsorbed CO and CH_4_ may react with NH_4_^+^ and form substances like HCN and formamide. The released fluid carrying HCN and formamide rises from an environment of relatively low pH into hydrated mantle of high pH. At the high pH, HCN monomers may form oligomers. HCN and formamide may form amino acids as well as purine and pyrimidine bases. Formamide may also form carbohydrates, which, together with the nitrogen bases may form nucleosides. These may, in turn, be phosphorylated by pyrophosphate or trimetaphosphate present in the system (based on the data by Fryer *et al.*, 1999).

Organic precursor molecules such as NH_3_, CO, and HCN are strongly adsorbed on secondary minerals like smectites and zeolites during seawater circulation at the slightly acidic pH of basalts of oceanic crust (Fripiat *et al.*, 1972; Gualtieri, 2000; Colín-García *et al.*, 2010). Once adsorbed, these molecules will be carried passively for perhaps millions of years along with the oceanic plate toward a subduction zone, as illustrated by the adsorption of methane, ethane, and propane to deep sea clays and their desorption by the addition of strong base (Hinrichs *et al.*, 2006). Convergent margins in the form of plate tectonic subduction zones are the most dynamic regions on Earth. The Mariana forearc in the western Pacific Ocean, where the Pacific plate subducts beneath the Philippines plate, is a non-accretionary forearc with numerous serpentine-brucite and carbonate mud volcanoes next to a deep ocean trench (Fig. 4). Near the Mariana trench, that is, at a lateral distance of 48–54 km from the maximum depth of the trench into the overriding Philippines plate, the upwelling pore waters of the Mariana forearc have pH of 10.7 and are less saline than the ambient seawater, because the waters originate by dehydration of the subducting Pacific slab at temperatures of 300–375 °C (Alt & Shanks, 2006; Mottl, 2009). These proximal springs form chimneys of the mineral brucite on the seafloor. Farther from the trench (70–90 km lateral distance), the fluid chemistry changes abruptly and the waters have pH 12.5 and are more concentrated with respect to dissolved inorganic species relative to seawater (Mottl, 2009). These distal springs form chimneys of aragonite and calcite. Serpentines are thus permeated by carbonate-rich, high-pH hydrothermal solutions at medium temperature (100–300 °C). The reason that the fluids close to the trench have a pH of about 10.7 is because of the consumption of H^+^ during serpentinization and brucite formation of primary silicate minerals (Holm & Neubeck, 2009). Mg(OH)_2_ is, in fact, excellent at buffering pH at alkaline conditions and has been used for that purpose in prebiotic peptide synthesis experiments (Huber *et al.*, 2003). However, the pH of 12.5 of the distal pore fluids requires an additional explanation, which is still under debate.

## Discussion

### The fossil record

It is believed that biological processes started to affect Earth’s surface mineralogy by 3.85–3.6 Ga (Hazen *et al.*, 2008). The Archean sedimentary record indicates that areas of shallow water on continental crust existed already by 3.6 Ga (Nisbet, 1987), and serpentine mud volcanoes similar to those of the Mariana forearc occur at Isua, Greenland, and have been dated to early Archean (3.81–3.70 Ga) (Pons *et al.*, 2011). Parts of the shallow seas were floored by evaporites, which show that the early ocean was not fresh: Its composition was in part controlled by hydrothermal circulation through spreading-centers consisting of komatiitic lavas (>18% MgO) (Nisbet, 1987; Schoonen *et al.*, 2004). Magnesite deposits that occur in Archaean strata are of controversial origin, although Nisbet (1987) suggests that they may be a variety of evaporite precipitated from fluids rich in magnesium and carbonate. However, the magnesites that exist in Isua are suggested to be precipitates from carbonate-rich fluids in subduction zones (Dymek *et al.*, 1988; Pons *et al.*, 2011).

Another group of evaporitic minerals are borates, which may be either marine or geothermal (‘non-marine’). Borate is rapidly taken up from seawater during low-temperature alteration of the oceanic crust (Palmer, 1991; Palmer & Swihart, 1996). It is enriched in serpentinites and basalts altered by seawater at relatively low temperatures (Bonatti *et al.*, 1984; Seyfried *et al.*, 1984). At 150 °C and below, boron is removed from seawater and is incorporated into alteration mineral phases like celadonite and Mg-smectite (Alt, 1995). A complete balance of the ocean boron budget requires deeply subducted boron to be returned efficiently to the oceans via arc volcanism. Several lines of evidence indicate that most of the boron is recycled from the subducted slab at convergent margins (You *et al.*, 1993), like the Marianas and the Lesser Antilles arcs (Mottl, 1992; Stout *et al.*, 2009).

Grew *et al.* (2011) reviewed the history and evolution of boron minerals to evaluate whether borate minerals could have been present to stabilize pentose sugars on the early Earth. The minerals that were primarily discussed in their paper were chosen on the basis of the experiments by Ricardo *et al.* (2004), in which ribose was stabilized in the presence of the marine evaporitic borate minerals ulexite (NaCaB_5_O_9_·8H_2_O), kernite (Na_2_B_4_O_7_), or colemanite (Ca_2_B_6_O_11_·5H_2_O). Grew *et al.* (2011) concluded that although there is no firm evidence that any of these studied minerals was present prior to 3.7 Ga, there could have been sufficient B for stabilizing ribose in the ocean if plate tectonics were active. Because the geological evidences known today support an initiation of plate tectonics on Earth in the Hadean eon, or before about 4 Ga ago (Harrison, 2009), a scenario with deeply subducted boron returned efficiently toward the seafloor via subduction of oceanic slabs and arc volcanism is highly likely. The oldest reported colemanite and ulexite deposits are only 330 Ma, and the oldest reported kernite just 19 Ma. However, 2.4–2.1 Ga borate deposits with an evaporitic origin exist in the Liaoning and Jilin Provinces, China. The borate mineralogy here is dominated by the magnesium borate minerals szaibelyite (Mg_2_B_2_O_5_·H_2_O), suanite (Mg_2_B_2_O_3_), with minor ludwigite ((Fe,Mg)_4_Fe_2_B_2_O_7_) associated with a sequence of metasediments and metavolcanics (Peng & Palmer, 2002). Suanite and szaibelyite are believed to have formed from dehydration of original hydrated borates like inderite (Mg_2_B_6_O_11_·15H_2_O). Geological, geochemical, and boron isotope studies indicate that these borates are metamorphosed evaporites of geothermal brines rich in B and Mg. The tectonic evolution of this area started with subduction of oceanic crust below an Archean craton, continental collision, and development of extensional basins. Geothermal fluids associated with the tectonic activity leached the oceanic crust that was in the process of subduction for enriched components, like boron and magnesium, and were channeled along fractures and faults through the overriding plate, in analogy to the processes in the Mariana and Lesser Antilles arc systems.

### Modern convergent margins

Serpentine-brucite type mud volcanism in convergent margin settings is not merely a local curiosity of the Mariana system but occurs worldwide and have done so through Earth’s history (Salisbury *et al.*, 2002; Hyndman & Peacock, 2003; Pons *et al.*, 2011). The forearc mud volcanoes are fed by a rising column of serpentinized peridotite that is mobilized by water and volatiles from the subducting plate. The mud volcanoes produced in this way accumulate large amounts of secondary minerals and can be big – about 2 km high and 50 km across. The Mariana forearc is of particular interest in the context of prebiotic organic synthesis because it is non-accretionary with respect to ocean floor sediments and the interaction between water and mafic rocks is not obscured by other processes. The boron concentration of pore water from ODP Site 1200 on the summit of the South Chamorro Seamount in the Mariana convergent margin has been shown to be 4 mmol kg^−1^ compared to 0.4 mmol kg^−1^ in seawater (Mottl *et al.*, 2003). Notably, the highest reported B concentration in geothermal springs of the St. Lucia island of the Lesser Antilles convergent margin is 135 mmol kg^−1^ (Stout *et al.*, 2009). Therefore, these kind of systems are probably the best modern analogs to the fossil environments in Liaoning and Jilin. The sedimentation rate of pelagic material is slow and ‘contamination’ by both biogenic and minerogenic particles is at a minimum. Interstitial fluids of pH 12.5 associated with serpentinized mud are enriched in dissolved components like carbonate, light hydrocarbons, borate and ammonia (Mottl *et al.*, 2003).

Phosphates are scavenged from seawater by ridge as well as ridge-flank hydrothermal activity and are accumulated in oceanic lithosphere in analogy to borates. Wheat *et al.* (1996) have estimated that ridge-axis and ridge-flank hydrothermal processes in combination remove about 50% of the global input of dissolved phosphorus from rivers into oceanic lithosphere. The occurrence of phosphate minerals is generally considered rare in ultramafic rocks. However, Chopin *et al.* (2001) report on the occurrence of raadeite (Mg_7_(PO_4_)_2_(OH)_8_) and other Mg-phosphates in nodules of apatite of the Tingelstadtjern serpentinite body, Norway.

In subduction zones, distillation, compaction, and smectite-to-illite transition are likely to be responsible for the liberation of organic precursor molecules. Because the fluids also carry the particulate matter that becomes the ‘serpentinite mud’ volcanoes, different types of active mineral surfaces are carried along. Analyses of the pore fluid composition show that the ultramafic rocks of the overriding Philippines plate in the Mariana system have reacted with a fluid derived from dehydration of the subducted slab of the Pacific plate, and not by seawater (Mottl, 1992). Subduction of oceanic lithosphere cools the overlying forearc close to the trench, so that the forearc mantle rocks get exceptionally cool, that is, ∼100 °C (Hyndman & Peacock, 2003). The alkaline pH of these subduction environments releases molecules like HCN and allows prebiotic synthesis of amino acids, carbohydrates, and heterocyclic nitrogen bases, etc. (Holm & Neubeck, 2009). The occurrence of cyanides in hydrothermal fluids of convergent margins has so far been reported from the Kurile Islands and the Kamchatka Peninsula (Mukhin, 1974; Mukhin *et al.*, 1978). The synthesis of these prebiotic key compounds and their stability are promoted by the prevalent moderate temperatures, as shown by the experiments by Ozawa *et al.* (2004) and LaRowe & Regnier (2008) (100–130 °C and 0–250 °C, respectively). Released fluid carrying HCN and formamide can be expected to rise from an environment of relatively low pH into hydrated mantle of high pH. At the high pH, HCN monomers may form oligomers. HCN and formamide may form amino acids as well as purine and pyrimidine bases. Formamide may also form carbohydrates, which may merge with nitrogen bases and form nucleosides. These may, in turn, be phosphorylated to nucleotides by pyrophosphate or trimetaphosphate present in the system. The nucleotides – the constituents of RNA – may eventually hybridize with the secondary DLH and in this way be stabilized for further prebiotic reactions in the interlayer space of the DLH.

## Concluding Remarks

John Maddox writes in a note in his book ‘What Remains to be Discovered’: ‘The close similarity of the chemical structure of the RNA molecules of the ribosomes from organisms of the same class is persuasive evidence of the antiquity of ribosomes in the evolution of living things’ (Maddox, 1999). Obviously, the ribosome is one of life’s most ancient machines. Magnesium is an essential element for any living organism today and has, no doubt, been so through life’s entire history on Earth. The fact that the biogeochemistry of magnesium is intimately coupled to that of phosphorus and nucleotides via the chemistries of boron and carbohydrates, indicates that it also had a key position in the prebiotic processes leading to the origin of life. Anyone who favors the notion that a pre-RNA world has existed must take its requirement for divalent magnesium of ribozymes and early enzymes like PPases for function into consideration. The major reason for its importance is that the properties of divalent magnesium are generally different from those of all other cations. Divalent magnesium has the largest hydrated radius of any common cation, but at the same time the ionic radius is among the smallest. Magnesium has always been abundant on Earth in the form of both monovalent (Mg(OH)^+^ and divalent cations (Mg^2+^) as well as hydrated complexes and secondary mineral phases in weathered seafloor. Borates and phosphates are accumulated in the same type of settings. Pyrophosphate and trimetaphosphate that can be active in prebiotic processes may be formed in environments of high pH and low water activity, like in forearc mantle rocks. Taken together, these circumstances point to the oceanic lithosphere as a likely environment for the initiation and propagation of life processes, particularly those parts of the ocean floor that were in the early phase of subduction at convergent margins.

## References

[b1] Alt JC, Humphris SE, Zierenberg RA, Mullineaux LS, Thomson RE (1995). Subseafloor processes in mid-ocean ridge hydrothermal systems. Seafloor Hydrothermal Systems; Physical, Chemical, Biological, and Geological Interactions.

[b2] Alt JC, Shanks WC (2006). Stable isotope compositions of serpentinite seamounts in the Mariana forearc: serpentinization processes, fluids sources and sulfur metasomatism. Earth and Planetary Science Letters.

[b3] Amaral AF, Marques MM, da Silva JAL, Fraústo da Silva JJR (2008). Interactions of D-ribose with polyatomic anions, and alkaline and alkaline-earth cations: possible clues to environmental synthesis conditions in the pre-RNA world. New Journal of Chemistry.

[b4] Anastassopoulou J (2003). Metal-DNA interactions. Journal of Molecular Structure.

[b5] Arrhenius GO, Sales B, Mojzsis S, Lee T (1997). Entropy and charge in molecular evolution – the case of phosphate. Journal of Theoretical Biology.

[b6] Baykov AA, Bakuleva NP, Rea PA (1993). Steady-state kinetics of substrate hydrolysis by vacuolar H^+^-pyrophosphatase: a simple three-state model. European Journal of Biochemistry.

[b7] Belogurov GA, Malinen AM, Turkina MV, Jalonen U, Rytkönen K, Baykov AA, Lahti R (2005). Membrane-bound pyrophosphatase of *Thermotoga maritima* requires sodium for activity. Biochemistry.

[b8] Benner SA (2004). Understanding nucleic acids using synthetic chemistry. Accounts of Chemical Research.

[b9] Bereźniak T, Zahran M, Imhof P, Jäschke A, Smith JC (2010). Magnesium-dependent active-site conformational selection in the Diels-Alderase ribozyme. Journal of the American Chemical Society.

[b10] Blankenship RE (2001). Molecular evidence for the evolution of photosynthesis. Trends in Plant Science.

[b11] Boesenberg JS, Hewins RH (2010). An experimental investigation into the metastable formation of phosphoran olivine and pyroxene. Geochimica et Cosmochimica Acta.

[b12] Bolaños L, Lukaszewski K, Bonilla I, Blevins D (2004). Why boron?. Plant Physiology and Biochemistry.

[b13] Bonatti E, Lawrence JR, Morandi N (1984). Serpentinization of oceanic peridotites: temperature dependence of mineralogy and boron content. Earth and Planetary Science Letters.

[b14] Cech TR (2002). Ribozyme mechanisms and folding; ribozymes, the first 20 years. Biochemical Society Transactions.

[b15] Cech TR, Zaug AJ, Grabowski PJ (1981). Invitro splicing of the ribosomal-RNA precursor of the *Tetrahymena*-involvement of a guanosine nucleotide in the excision of the intervening sequence. Cell.

[b16] Chopin C, Ferraris G, Prencipe M, Brunet F, Medenbach O (2001). Raadeite, Mg_7_(PO4)_2_(OH)_8_: a new dense-packed phosphate from Modum (Norway). European Journal of Mineralogy.

[b17] Choy JH, Kwak SY, Park JS, Jeong YJ, Portier J (1999). Intercalative nanohybrids of nucleoside monophosphates and DNA in layered metal hydroxide. Journal of the American Chemical Society.

[b18] Choy JH, Oh JM, Park M, Sohn KM, Kim JW (2004). Inorganic-biomolecular hybrid nanomaterials as a genetic molecular code system. Advanced Materials.

[b19] Colín-García M, Ortega-Gutiérrez F, Ramos-Bernal S, Negrón-Mendoza A (2010). Heterogenous radiolysis of HCN adsorbed on a solid surface. Nuclear Instruments and Methods in Physics Research A.

[b20] Delacour A, Früh-Green GL, Bernasconi SM, Schaeffer P, Kelley DS (2008). Carbon geochemistry of serpentinites in the Lost City hydrothermal system (30°N, MAR). Geochimica et Cosmochimica Acta.

[b21] Delidovich IV, Simonov AN, Pestunova OP, Parmon VN (2009). Catalytic condensation of glycolaldehyde and glyceraldehyde with formaldehyde in neutral and weakly alkaline aqueous media: kinetics and mechanism. Kinetics and Catalysis.

[b22] Dymek RF, Brothers SC, Schiffries CM (1988). Petrogenesis of ultramafic rocks from the 3800 Ma Isua Supracrustal Belt, West Greenland. Journal of Petrology.

[b23] El Goresy A, Ramdohr P, Taylor LA (1971). The geochemistry of the opaque minerals in Apollo 14 crystalline rocks. Earth and Planetary Science Letters.

[b24] Etaix E, Orgel LE (1978). Phosphorylation of nucleosides in aqueous solution using trimetaphosphate: formation of nucleoside triphosphates. Journal of Carbohydrates, Nucleosides, and Nucleotides.

[b25] Ferris JP (1984). The chemistry of life’s origin. Chemical and Engineering News.

[b26] Ferris JP (2005). Catalysis and prebiotic synthesis. Reviews in Mineralogy and Geochemistry.

[b27] Ferris JP, Usher DA, Zubay G (1988). Origins of life. Biochemistry.

[b28] Flanagan G, Ahmed SN, Shevlin PB (1992). Formation of carbohydrates in the reaction of atomic carbon with water. Journal of the American Chemical Society.

[b29] Fox GE, Naik AK, de Pouplane LR (2004). The evolutionary history of the translation machinery. The Genetic Code and the Origin of Life.

[b30] Freisinger E, Sigel RKO (2007). From nucleotides to ribozymes – a comparison of their metal ion binding properties. Coordination Chemistry Reviews.

[b31] Fripiat JJ, Poncelat G, van Assche AT, Mayaudon J (1972). Zeolites as catalysts for the synthesis of amino acids and purines. Clays and Clay Minerals.

[b500] Fryer P, Wheat CG, Mottl MG (1999). Mariana blueschist mud volcanism: implications for conditions within the subduction zone. Geology.

[b32] Gedulin B, Arrhenius G, Bengtson S (1994). Sources and geochemical evolution of RNA precursor molecules: the role of phosphate. Early Life on Earth.

[b33] Gilbert W (1986). Origin of life: the RNA world. Nature.

[b34] Gonella NC, Nakanishi H, Holtwick JB, Horowitz DS, Nanamori K, Leonard NJ, Roberts JD (1983). Studies of tautomers and protonation of adenine and its derivatives by nitrogen-15 nuclear magnetic resonance spectroscopy. Journal of the American Chemical Society.

[b35] Grew ES, Bada JL, Hazen RM (2011). Borate minerals and origin of the RNA World. Origins of Life and Evolution of the Biospheres.

[b36] Grubbs RD (2002). Intracellular magnesium and magnesium buffering. BioMetals.

[b37] Gualtieri AF (2000). Study of NH_4_^+^ in the zeolite phillipsite by combined synchrotron powder diffraction and IR spectroscopy. Acta Chrystallographica.

[b38] Harrison TM (2009). The Hadean crust: evidence from >4 Ga zircons. Annual Reviews in Earth and Planetary Sciences.

[b39] Hazen RM, Papineau D, Bleeker W, Downs RT, Ferry JM, McCoy TJ, Sverjensky DA, Yang H (2008). Mineral evolution. American Mineralogist.

[b40] Hermes-Lima M, Vieyra A (1992). Pyrophosphate synthesis from phospho(enol)pyruvate catalyzed by precipitated magnesium phosphate with “enzyme-like” activity. Journal of Molecular Evolution.

[b41] Herrero LA, Terrón A (1998). Complexation in solution of magnesium(II) and cobalt(II) with purine 5′-monophosphates and pyrimidine 5′-monophosphates: a potentiometric and calorimetric study. Polyhedron.

[b42] Hinrichs KU, Hayes JM, Bach W, Spivack AJ, Hmelo LR, Holm NG, Johnson CG, Sylva SP (2006). Biological formation of ethane and propane in the deep marine subsurface. Proceedings of the National Academy of Sciences of the United States of America.

[b43] Holm NG, Baltscheffsky H (2011). Links between hydrothermal environments, pyrophosphate, Na^+^, and early evolution. Origins of Life and Evolution of the Biospheres.

[b44] Holm NG, Neubeck A (2009). Reduction of nitrogen compounds in oceanic basement and its implications for HCN formation and abiotic organic synthesis. Geochemical Transactions.

[b45] Holm NG, Neubeck A, Ivarsson M, Konn C, Basiuk VA (2010). Abiotic organic synthesis beneath the ocean floor. Astrobiology: Emergence, Search and Detection of Life.

[b46] Hsiao CL, Williams LD (2009). A recurrent magnesium-binding motif provides a framework for the ribosomal peptidyl transferase center. Nucleic Acids Research.

[b47] Hsiao CL, Mohan S, Kalahar BK, Williams LD (2009). Peeling the onion: ribosomes are ancient molecular fossils. Molecular Biology and Evolution.

[b48] Huber C, Eisenreich W, Hecht S, Wächtershäuser G (2003). A possible primordial peptide cycle. Science.

[b49] Hyndman RD, Peacock SM (2003). Serpentinization of the forearc mantle. Earth and Planetary Science Letters.

[b50] Joyce GF (1989). RNA evolution and the origin of life. Nature.

[b51] Kalliney SY, Griffiths EJ, Martin D (1972). Cyclophosphates. Topics in Phosphate Chemistry.

[b52] Karl DM, Tien G (1992). MAGIC: a sensitive and precise method for measuring dissolved phosphorus in aquatic environments. Limnology and Oceanography.

[b53] Kehres DG, Maguire ME (2002). Structure, properties and regulation of magnesium transport proteins. BioMetals.

[b54] Klein DJ, Moore PB, Steitz TA (2004). The contribution of metal ions to the structural stability of the large ribosomal subunit. RNA.

[b55] Kongshaug KO, Fjellvåg H, Lillerud KP (2000). Synthesis and crystal structure of the hydrated magnesium diphosphate Mg_2_P_2_O_7_^.^3.5H_2_O and its high temperature variant Mg_2_P_2_O_7_^.^H_2_O. Solid State Science.

[b56] Kruger K, Grabowski PJ, Zaug AJ, Sands J, Gottschling DE, Cech TR (1982). Self-splicing RNA: autoexcision and autocyclization of the ribosomal RNA intervening sequence of *Tetrahymena*. Cell.

[b57] LaRowe DE, Helgeson HC (2006). The energetics of metabolism in hydrothermal systems: calculation of the standard molal thermodynamic properties of magnesium-complexed adenosine nucleotides and NAD and NADP at elevated temperatures and pressures. Thermochimica Acta.

[b58] LaRowe DE, Regnier P (2008). Thermodynamic potential for the abiotic synthesis of adenine, cytosine, guanine, thymine, uracil, ribose, and deoxyribose in hydrothermal systems. Origins of Life and Evolution of the Biospheres.

[b59] Li Q, Ricardo A, Benner SA, Winefordner JD, Powell DH (2005). Desorption/ionization on porous silicon mass spectrometry studies on pentose-borate complexes. Analytical Chemistry.

[b60] Lindh U, Selinus O, Alloway B, Centeno JA, Finkelman RB, Fuge R, Lindh U, Smedley P (2005). Biological functions of the elements. Essentials of Medical Geology; Impacts of the Natural Environment on Public Health.

[b61] Luoto H, Belogurov GA, Baykov AA, Lahti R, Malinen AM (2011). Na^+^-translocating membrane pyrophosphatases are widespread in the microbial world and evolutionary precede H^+^-translocating pyrophosphatases. Journal of Biological Chemistry.

[b62] Maddox J (1999). What Remains to Be Discovered: Mapping the Secrets of the Universe, the Origins of Life, and the Future of Human Race.

[b63] Maguire ME, Cowan JA (2002). Magnesium chemistry and biochemistry. BioMetals.

[b64] Malinen AM, Baykov AA, Lahti R (2008). Mutual effects of cationic ligands and substrate activity of the Na^+^-transporting pyrophosphatase of *Methanosarcina mazei*. Biochemistry.

[b65] Mellersh AR, Smith PM (2010). The alkaline world and the origin of life. Journal of Cosmology.

[b66] Mottl MJ (1992). Pore waters from serpentinite seamounts in the Mariana and Izu-Bonin forearc, Leg 125: evidence for volatiles from the subducting slab. Proceedings of the Ocean Drilling Program, Scientific Results.

[b67] Mottl MJ (2009). Highest pH?. Geochemical News.

[b68] Mottl MJ, Komor SC, Fryer P, Moyer CL (2003). Deep-slab fluids fuel extremophilic *Archaea* on a Mariana forearc serpentinite mud volcano: Ocean Drilling Program Leg 195. Geochemistry, Geophysics, Geosystems.

[b69] Mukhin LM (1974). Evolution of organic compounds in volcanic regions. Nature.

[b70] Mukhin LM, Bondarev VB, Safonova EN (1978). The role of volcanic processes in the evolution of organic compounds on the primitive Earth. Modern Geology.

[b71] Mulkidjanian AY, Galperin MY, Makarova KS, Wolf YI, Koonin EV (2008). Evolutionary primacy of sodium energetics. Biology Direct.

[b72] Neubeck A (2011).

[b73] Nisbet EG (1987). The Young Earth: An Introduction to Archaean Geology.

[b74] Olson JM (2006). Photosynthesis in the Archean Era. Photosynthesis Research.

[b75] Ozawa K, Nemoto A, Imai EI, Honda H, Hatori K, Matsuno K (2004). Phosphorylation of nucleotide molecules in hydrothermal environments. Origins of Life and Evolution of the Biosphere.

[b76] Palmer MR (1991). Boron isotope systematics of hydrothermal fluids and tourmalines: a synthesis. Chemical Geology (Isotope Geoscience Section).

[b77] Palmer MR, Swihart GH, Grew ES, Anovitz LM (1996). Boron isotope geochemistry: an overview. Reviews in Mineralogy Volume 33: Boron; Mineralogy, Petrology and Geochemistry.

[b78] Panagiotopoulos C, Sempéré R (2005). Analytical methods for the determination of sugars in marine samples: a historical perspective and future directions. Limnology and Oceanography: Methods.

[b79] Pasek MA (2008). Rethinking early Earth phosphorus geochemistry. Proceedings of the National Academy of Sciences of the United States of America.

[b80] Pasek MA, Lauretta DS (2005). Aqueous corrosion of phosphide minerals from iron meteorites: a highly reactive source of prebiotic phosphorus on the surface of the early Earth. Astrobiology.

[b81] Pasek MA, Dworkin JP, Lauretta DS (2007). A radical pathway for phosphorylation during schreibersite corrosion with implications for the origin of life. Geochimica et Cosmochimica Acta.

[b82] Pasek MA, Kee TP, Bryant DE, Pavlov AA, Lunine JI (2008). Production of potentially condensed phosphates by phosphorus redox chemistry. Angewandte Chemie, International Edition.

[b83] Pauly H (1969). White cast iron with cohenite, schreibersite, and sulfides from Tertiary basalts on Disko, Greenland. Bulletin of the Geological Society of Denmark.

[b84] Peng QM, Palmer MR (2002). The Paleoproterozoic Mg and Mg-Fe borate deposits of Liaoning and Jilin Provinces, northeast China. Economic Geology.

[b85] Petrov AS, Bowman JC, Harvey SC, Williams LD (2011). Bidentate RNA-magnesium clamps: on the origin of the special role of magnesium in RNA folding. RNA.

[b86] Pokrovsky OS, Schott J, Castillo A (2005). Kinetics of brucite dissolution at 25°C in the presence of organic and inorganic ligands and divalent metals. Geochimica et Cosmochimica Acta.

[b87] Pons ML, Quitté G, Fujii T, Rosing MT, Reynard B, Moynier F, Douchet C, Albarède F (2011). Early Archean serpentine mud volcanoes at Isua, Greenland, as a niche for early life. Proceedings of the National Academy of Sciences of the United States of America.

[b88] Prieur BE (2001). Étude de l’activité prébiotique potentielle de l’acide borique. Acad. Sci. Paris, Chimie/Chemistry.

[b89] Prieur BE (2009). Phosphorylation of ribose in the presence of borate salts. Origins of Life and Evolution of the Biospheres.

[b90] Rauchfuss H (2008). Chemical Evolution and the Origin of Life.

[b91] Ricardo A, Carrigan MA, Olcott AN, Benner SA (2004). Borate minerals stabilize ribose. Science.

[b92] Russell MJ, Hall AJ (1997). The emergence of life from iron monosulphide bubbles at a submarine hydrothermal redox and pH front. Journal of the Geological Society.

[b93] Saladino R, Barontini M, Cossetti C, Di Mauro E, Crestini C (2011). The effect of borate minerals on the synthesis of nucleic acid bases, amino acids and biogenic carboxylic acids from formamide. Origins of Life and Evolution of the Biospheres.

[b94] Sales BC, Chakoumakos BC, Boatner LA, Ramey JO (1993). Structural properties of the amorphous phases produced by heating crystalline MgHPO_4_^.^3H_2_O. Journal of Non-Crystalline Solids.

[b95] Salisbury MH, Shinohara M, Richter C, Araki E, Barr SR, D’Antonio M, Dean SM, Diekmann D, Edwards KM, Fryer PB, Gaillot GJ, Hammon WS, Hart D, Januszczak N, Komor SC, Kristensen MB, Lockwood JP, Mottl MJ, Moyer CL, Nakahigashi K, Savov IP, Su X, Wei KU, Yamada T (2002). Proceedings of the Ocean Drilling Program, Initial Reports.

[b96] Schoonen M, Smirnov A, Cohn C (2004). A perspective on the role of minerals in prebiotic synthesis. Ambio.

[b97] Schwartz AW, Buvet R, Ponnamperuma C (1971). Phosphate: solubilization and activation on the early Earth. Chemical Evolution and the Origin of Life.

[b98] Schwartz AW (2006). Phosphorus in prebiotic chemistry. Philosophical Transactions of the Royal Society B.

[b99] Seel F, Klos KP, Schuh J (1985). Hydrothermale Kondensation von Magnesium-hydrogenphosphaten zu Magnesiumdiphosphaten. Naturwissenschaften.

[b100] Seel F, Klos KP, Rechtenwald D, Schuh J (1986). Non-enzymatic formation of condensed phosphates under prebiotic conditions. Zeitung der Naturforschung Section B.

[b101] Seyfried WE, Janecky DR, Mottl MJ (1984). Alteration of the oceanic crust: implications for geochemical cycles of lithium and boron. Geochimica et Cosmochimica Acta.

[b102] Sreedhara A, Cowan JA (2002). Structural and catalytic roles for divalent magnesium in nucleic acid biochemistry. BioMetals.

[b103] Stout LM, Blake RE, Greenwood JP, Martini AM, Rose EC (2009). Microbial diversity of boron-rich volcanic hot springs of St. Lucia, Lesser Antilles. FEMS Microbiology Ecology.

[b104] Turian G, Rivara-Minten E (2001). Prebiotic phosphoramidation of nucleobases by Mg^2+^-triggered decyclization of trimetaphosphate. Archives des Sciences.

[b105] Ulff-Møller F (1985). Solidification history of the Kitdlit Lens: immiscible metal and sulphide liquids from a basaltic dyke on Disko, central West Greenland. Journal of Petrology.

[b106] Walter NG, Engelke DR (2002). Ribozymes: catalytic RNAs that cut things, make things, and do odd and useful jobs. Biologist.

[b107] Westheimer FH (1987). Why nature chose phosphates. Science.

[b108] Wheat CG, Feely RA, Mottl MJ (1996). Phosphate removal by oceanic hydrothermal processes; an update of the phosphorus budget of the oceans. Geochimica et Cosmochimica Acta.

[b109] Yamagata Y, Watanabe H, Saitoh M, Namba T (1991). Volcanic production of polyphosphates and its relevance to prebiotic evolution. Nature.

[b110] Yamagata Y, Inoue H, Inomata K (1995). Specific effect of magnesium ion on 2′,3′-cyclic AMP synthesis from adenosine and trimeta phosphate in aqueous solution. Origins of Life and Evolution of the Biosphere.

[b111] Yang LJ, Arora K, Beard WA, Wilson SH, Schlick T (2004). Critical role of magnesium ions in DNA polymerase β’s closing and active site assembly. Journal of the American Chemical Society.

[b112] You CF, Spivack AJ, Smith JH, Gieskes JM (1993). Mobilization of boron in convergent margins: implications for the boron geochemical cycle. Geology.

[b113] Yuasa S, Ishigami M (1977). Geochemically possible condensation of hydrogen cyanide in the presence of divalent metal compounds. Geochemical Journal.

[b114] de Zwart II, Meade SJ, Pratt AJ (2004). Biomimetic phosphoryl transfer catalysed by iron(II)-mineral precipitates. Geochimica et Cosmochimica Acta.

